# A Pitfall of Adrenal Hypoplasia Congenita

**DOI:** 10.1177/00099228231222714

**Published:** 2024-01-27

**Authors:** Jiro Abe, Junko Tsubaki, Kazuhiro Shimura, Tomonobu Hasegawa

**Affiliations:** 1 Department of Medicine, University of Cambridge, Cambridge, UK; 2 Department of Pediatrics, JCHO Hokkaido Hospital, Sapporo, Japan; 3 Department of Pediatrics, Keio University Hospital, Tokyo, Japan

**Educational Objectives**

- This case report serves as a reminder of the critical nature of adrenal insufficiency, particularly in neonates, and highlights the need for health care providers to be vigilant in recognizing its signs and symptoms even in the absence of known risk factors.

## Case Report

A male neonate, the first baby for Mother, was born with no significant familial or obstetric histories, including consanguineous marriage. He was delivered vaginally at term, weighing 3 kg and 50 cm of body height, in an obstetric clinic. He needed early milk feeding because of sustained low blood glucose levels within the range of 2.2 to 3.3 mmol/L. He was transferred to our neonatal intensive care unit due to his difficulty of oral-feeding and maintenance of blood glucose 1 day after birth.

## Hospital Course

**Figure 1.**
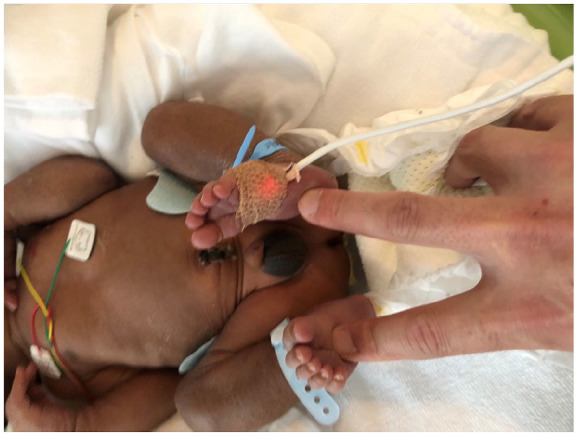
Patient’s appearance. *Note.* Hyperpigmentation is global and most noticeable on the scrotum.

**Figure 2.**
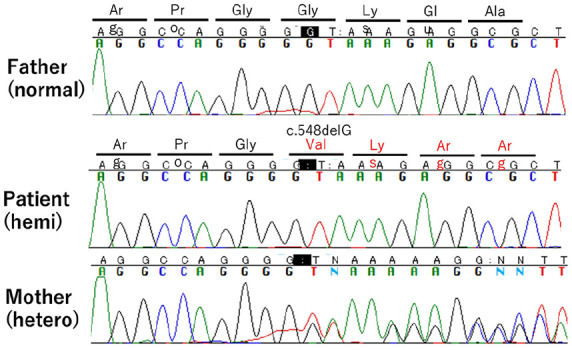
Sanger sequencing. *Note.*Patient’s chromatogram (middle) showed the variant of NROB1 (NM_000475) as c.548delG: p. Gly183Val fs*81. This variant turned out to derive from Mother (bottom), while Father (top) had no deletion of the codon G (Black).

The neonate was generally unwell with poor oral intake, exhaustion, and poor muscle tone. Notable symptoms included recurrent vomiting and failure to thrive during the first week of life, followed by systemic subcutaneous hyperpigmentation (Figure 1). He remained unwell despite treatments of glucose infusion and tube-feeding. The admission investigation of blood tests showed serum sodium 135 mEq/L, potassium 4.8 mEq/L, and glucose 3.0 mmol/L. One week after birth, his biochemical findings revealed the onset of salt-losing syndrome: serum sodium 123 mEq/L, potassium 6.4 mEq/L, glucose 3.5 mmol/L, pH 7.34, bicarbonate 23.5 mEq/L. His ACTH level proved 818 pg/mL while the level of cortisol was 1 mcg/dL, which shows adrenal insufficiency (Table 1). Ultrasonography and magnetic resonance imaging showed agenesis of adrenal glands (data-not-shown). These clinical findings lead to the diagnosis of adrenal hypoplasia congenita (AHC). Based on parents’ consent to have genetic tests, the genetic panel analyses for AHC-related genes including *AAAS*, *ABCD1*, *CDKN1C*, *CYB5A*, *CYP11A1*, *CYP11B1*, *CYP11B2*, *CYP17A1*, *GPX1*, *HSD17B3*, *HSD3B2*, *MC2R*, *MCM4*, *MRAP*, *NNT*, *NROB1*, *ROR*, *STAR*, *TXNRD2*, and *SAMD9* were performed, which showed the hemizygous variant of *NROB1* (c.548delG, p. Gly183Val fs*81). The chromatograms of the patient and his parents by Sanger sequence revealed the variant was derived from Mother (Figure 2). Analysis of urine steroid profile revealed low urinary Δ5 value strongly suggesting AHC or congenital adrenal lipoid hyperplasia (data-not-shown). Analysis of long chained fatty acids was omitted because the genetic panels found *ABCD1* gene sequence normal.

**Table 1.** Laboratory findings.

Hormonal levels		Case	Normal range
ACTH	pg/mL	818	5-77
Cortisol	μg/dL	1	3.7-19.4
17-OHP	ng/mL	1.17	<8
DHEA-S	ng/mL	111	240-5370
11-OHCS	μg/dL	3.4	7.0-23.0
Testosterone	ng/dL	37.7	0-177
Aldosterone	pg/mL	4	4.0-82.1
Renin	pg/mL	210.8	2.21-39.5

Abbreviations: ACTH: Adrenocorticotropic Hormone; DHEA-S: Dehydroepiandrosterone Sulfate; 17-OHP: 17-Hydroxyprogesterone; 11-OHCS: 11-Hydroxycorticosteroids.

Treatment for adrenal insufficiency was initiated with 2 bolus intravenous infusions of 10 mg/kg hydrocortisone, followed by maintenance therapy with 10 mg/kg daily, which was later switched to 3.5 mg/kg oral therapy. Fludrocortisone was added from 0.05 mg as an initial dose and increased to 0.1 mg daily to cover mineralocorticoid needs 3 days after hydrocortisone therapy. The patient responded positively to the treatment, with an improvement in clinical symptoms, including the resolution of hyperpigmentation. ACTH level turned 63.9 pg/mL 1 month after birth. Long-term monitoring of reproductive function as well as endocrine health issues will be planned besides genetic counseling for family members because of DAX-1 related conditions.

## Discussion of Case and Literature

DAX-1 (Dosage-sensitive sex reversal-Adrenal hypoplasia congenita critical region on the X chromosome 1, genetically named as *NR0B1*) plays a main role in human development of adrenal glands and reproductive organ. Loss-of-function of *NR0B1* gene is responsible for AHC, which typically affects male children as primary adrenal insufficiency especially during the neonatal period.^1,2^ In addition, patients with AHC have hypogonadotropic hypogonadism leading to impaired spermatogenesis during puberty. Early genetic diagnosis can help reduce significant risks of morbidity and mortality, facilitate planning of hormone replacement therapies, and genetic counseling.

Clinicians should maintain a high level of clinical suspicion when encountering neonates or individuals with symptoms of adrenal insufficiency to ensure prompt diagnosis and intervention, especially in cases like AHC, which can have severe consequences if left untreated. Adrenal hypoplasia congenita is not detectable through routine newborn metabolic screening. This highlights the importance of clinical awareness and genetic testing when there are signs and symptoms indicative of adrenal insufficiency. Although AHC typically presents during the neonatal period, clinicians should be aware that late-onset cases have been reported. In such cases, a high degree of suspicion is required to make a diagnosis. The onset of salt-losing syndrome is a key indicator of AHC. As observed in this case, salt-losing symptoms often precede the diagnosis,^3^ and clinicians should be vigilant when they encounter patients with sustained hypoglycemia, failure to thrive, and hyperpigmentation. Recognizing these symptoms can lead to earlier diagnosis and intervention. The pathogenic variant which we reported here leads to loss of normal protein function either through protein truncation or non-sense mediated mRNA decay based on bioinformatics and in silico data.^4,5^ This is the first clinical report of the hemizygous variant of *NROB1* (c.548delG, p. Gly183Val fs*81).

## Final Diagnosis

AHC: *DAX-1* variant (c.548delG, p. Gly183Val fs*81).

## Conclusion

The prompt initiation of hormone replacement therapy was essential for the survival and ongoing health of the patient. This case underscores the critical role of genetic testing in facilitating early diagnosis and guiding the clinical management of AHC, ultimately improving patient outcomes.

## Author Contributions

JA designed and prepared the manuscript; KS performed genetic analysis, and JT and TH provided technical support and conceptual advice. All of the authors read and approved the final version of the manuscript.
